# Advances in Gene Therapy for Age-Related Macular Degeneration: A Narrative Review

**DOI:** 10.3390/jcm15083097

**Published:** 2026-04-18

**Authors:** Noor-Us-Sabah Ahmad, Tahreem A. Mir

**Affiliations:** 1 Department of Ophthalmology and Visual Sciences, Carver College of Medicine, University of Iowa, Iowa City, IA 52242, USA; noor-us-sabah-ahmad@uiowa.edu; 2 Institute for Vision Research, University of Iowa, Iowa City, IA 52242, USA

**Keywords:** gene therapy, age-related macular degeneration, neovascular age-related macular degeneration, viral vectors, vascular endothelial growth factor

## Abstract

Age-related macular degeneration (AMD) is the most common cause of blindness and vision impairment in individuals over 60 years of age in the United States (US). Despite this, current treatment options have limitations related to drug efficacy and durability. Gene therapy provides a potential solution by providing a more durable and longer- acting treatment option that can decrease treatment burden and improve long-term visual outcomes. This review presents the current treatment approaches, routes of administration, and vectors being investigated for gene therapy delivery in AMD. It also provides an update on the ongoing gene therapy clinical trials for dry and wet AMD. As these therapies advance into later-stage clinical trials, ophthalmologists need to be mindful of the many challenges pertaining to gene therapy delivery, including safety, limitations related to immunogenicity, long-term ocular and systemic side effects, and potential barriers to drug manufacturing and access. Continued efforts are required to improve precision, safety, and efficacy, including identifying the safest and most effective vectors and delivery routes, and minimizing potential adverse effects. In addition, guidelines need to be established to guide appropriate patient selection before gene therapy can be integrated into clinical practice.

## 1. Introduction

Approximately 20 million individuals in the United States (US) have age-related macular degeneration (AMD), and the prevalence is expected to increase with an aging population [1]. Globally, it is estimated that 1.85 million people are legally blind due to AMD, and another 6.23 million suffer from moderate or severe visual impairment from the disease [2]. Risk factors for AMD include smoking, increased age, hypercholesterolemia, and obesity [3]. Several genetic factors have been associated with the risk of disease development and progression. Human genome-wide association studies have identified about 34 genetic loci for AMD on at least 19 chromosomes [4]. The two major susceptibility genes for AMD include complement factor H (CFH) gene on chromosome 1 and ARMS2/HTRA1 gene on chromosome 10 [5].

Age-related macular degeneration can be broadly classified into non-neovascular (dry) or neovascular (wet) AMD. Non-neovascular AMD comprises 85 to 90% of AMD cases and is characterized by the presence of drusen, retinal pigment epithelium (RPE) changes, and eventually loss of photoreceptors and extensive outer retinal atrophy, called geographic atrophy (GA), that leads to severe vision loss [3,6]. Neovascular AMD (nvAMD) comprises 10–15% of AMD cases and is considered an advanced form of AMD that can cause severe vision loss without treatment. It is characterized by choroidal neovascularization, which can lead to subretinal or intraretinal fluid and submacular hemorrhage [3].

Over the last two decades, there have been marked advancements in the treatment of nvAMD. This is due to the development of intravitreal anti-angiogenic treatments that primarily target the vascular endothelial growth factor (VEGF) family of proteins [7]. Vascular endothelial growth factor induces endothelial migration and proliferation leading to neovascularization. The abnormal vessels generated by this process can leak and bleed, leading to vision loss [8]. Commercially available anti-VEGF agents cause regression of neovascularization by decreasing the levels of VEGF. Population-based cohort studies show that with timely and consistent treatment with anti-VEGF agents, 90% of eyes with nvAMD show stabilization or improvement in vision over time and only 6% of eyes develop severe vision loss (defined by loss of >15 EDTRS letters) [9]. However, in most patients, the excess production of VEGF is chronic, requiring frequent, often monthly intravitreal injections for a prolonged period of time to maintain visual acuity. This poses a significant treatment burden on the patient, injection fatigue, and eventual loss to follow-up. The need for frequent injections can also pose a substantial economic burden on patients and their caregivers, stemming not only from the cost of the medication itself but also from expenses related to office visits, travel time, and time off work. This can contribute to treatment non-adherence and potentially poor long-term visual outcomes [10,11,12]. A study evaluating data from 156,327 patients with nvAMD over six years in the Intelligent Research In Sight (IRIS) registry database showed that one in 9 patients were lost to follow-up and one in seven patients had nonpersistent follow-up [13]. Studies have shown that the initial visual acuity gains achieved through anti-VEGF treatment of nvAMD patients in clinical trials declined with less frequent follow-up over time [14,15]. Between 2011 and 2015, the annual Medicare expenditure was $1.3 billion for ranibizumab and $1.4 billion for aflibercept, with these two drugs accounting for approximately 12% of the Medicare Part B budget [16].

Treatment options for dry AMD are also limited. Before the recent Food and Drug Administration (FDA) approval of complement factor inhibitors, there were no treatments for dry AMD. The mainstay of therapy was lifestyle modification and supplementation of micronutrients (per the Age-Related Eye Disease Study or AREDS) to attempt to slow the progression of the disease [17]. Two complement factor inhibitors are currently approved in the US for geographic atrophy (GA) due to dry AMD, a C3 complement inhibitor called pegcetacoplan, and a C5 complement inhibitor called avacincaptad pegol. Intravitreal injections of these agents have been shown to reduce the rate of GA growth; however, no significant visual acuity benefits have been shown [18,19] and the need for frequent injections pose treatment and economic burdens similar to those associated with anti-VEGF agents. Other therapies that are being explored for AMD include autologous RPE and choroid grafts as well as somatic stem cell therapy, which have not yet entered the realm of clinical practice [20,21]. Recently, the advent of a subretinal photovoltaic implant has shown some visual benefit in eyes affected with central GA; however, this intervention does not prevent the development of GA itself or treat the underlying disease process [22].

Gene therapy provides a potential solution by providing a more durable and longer acting treatment option that can decrease treatment burden and improve long-term outcomes. It involves transferring genetic material, i.e., either DNA or RNA, into the cells of patients, thus prompting endogenous production of proteins that are either deficient or defective in disease states, or otherwise beneficial in treating the disease. Gene therapy for AMD has several potential benefits compared with conventional drug therapy. Unlike conventional therapy, which requires repeated treatments, gene therapy has the potential to act as a one-time treatment option by allowing the sustained intraocular production of proteins after a single administration. It may result in more stable disease control by providing continuous expression of therapeutic proteins and thus avoiding the pharmacokinetic trough that occurs with standard treatment. It can also avoid the potential of treatment failure related to patient adherence and loss to follow-up, as well as reduce cumulative risks and long-term costs. The eye is a promising target for gene therapy due to its unique immune privilege status, ability to administer therapy through direct routes, and a matched contralateral control [23]. This has been proven by the fact that the first FDA-approved gene therapy in the United States was voretigene neparvovec-rzyl (Luxturna), which was designed to treat inherited retinal blindness caused by mutations in the RPE-65 gene [24].

The purpose of this review is to summarize the current advances in gene therapy for AMD, with particular emphasis on genetic vectors, delivery approaches, drug safety and efficacy, clinical outcomes, and safety profiles. Additionally, this review seeks to contextualize current evidence within the evolving treatment landscape and highlight key challenges, limitations, and future directions for gene therapies for AMD. This review includes both completed and ongoing gene therapy trials for AMD. However, given the heterogeneity of study designs and the fact that many trials are ongoing or have not yet begun enrollment, a formal systematic review was not feasible. Accordingly, we present the current landscape as a narrative review.

## 2. Gene Therapy Approaches and Genetic Vectors

Approaches to gene therapy include: (1) gene replacement or augmentation, in which a normal copy of a defective or missing gene is introduced to restore production of the functional protein; (2) gene editing, which involves directly correcting the disease-causing mutation within the patient’s genetic material; (3) gene silencing, which suppresses expression of disease-causing gain-of-function or dominant-negative genes; (4) the “biofactory approach”, in which nonnative genes for desired therapeutic proteins are introduced into cells to modulate the disease course [25,26].

Vectors for gene therapy include viral and non-viral vectors. The most extensively studied viral vectors include adenovirus (AdV), adeno-associated virus (AAV), and lentivirus [7]. Various factors determine the choice of vector, such as tropism (affinity for specific tissue), payload capacity (the amount of genetic material that a viral vector can successfully carry), and safety profile including immunogenicity and genotoxicity. The most common viral vector used in the gene therapy of retinal diseases is AAV. It has excellent tissue-specific tropism, low immunogenicity, and low risk of genotoxicity. Adeno-associated virus can infect non-dividing cells, and its genetic material remains mostly episomal (does not integrate into the host cell’s genome). However, its use for certain purposes is limited by its small payload capacity [7,27]. Adenovirus can transport a substantially larger genetic payload, and like AAV, its genetic material remains episomal, and it can infect non-dividing cells. However, AdV is highly immunogenic, limiting its use [28]. Lentiviral vectors enable integration of the delivered genetic material into the host genome, enabling a sustained long-term response. They can deliver larger genes than AAV and are moderately immunogenic. However, they have a risk of genotoxicity (insertional oncogenesis) due to their integration into host DNA [7]. Non-viral vectors include naked nucleic acids (DNA or RNA), polymer-based vectors, lipid-based vectors (liposomes and lipid nanoparticles), and peptide- or protein-based carriers [25,27]. Even though non-viral vectors have a lower risk of toxicity and immunogenicity, their use is limited by reduced stability, lower specificity, and lower transfection efficiency.

## 3. Routes of Administration

**Figure 1:**
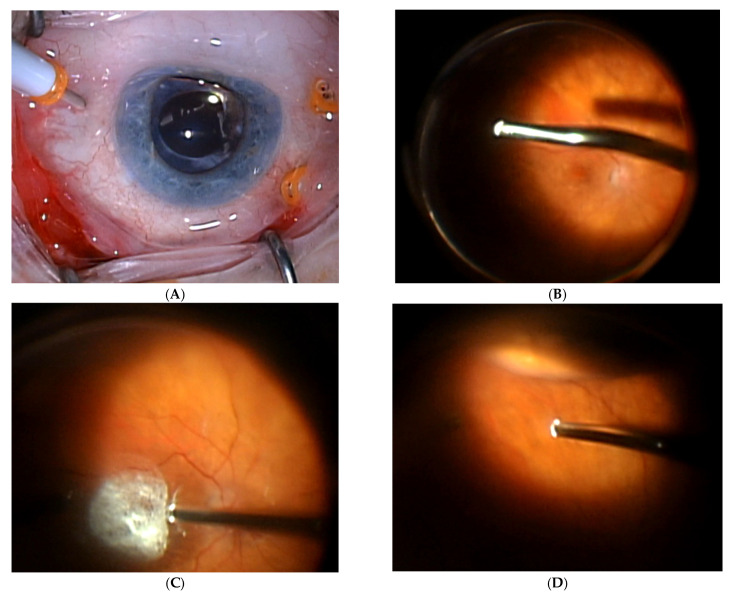

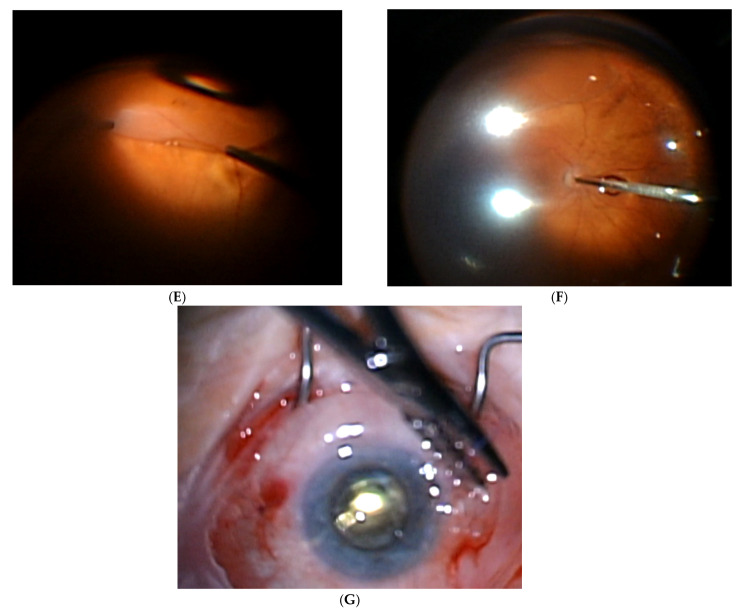
Surgical Technique Demonstrating Pars Plana Vitrectomy for Subretinal Gene Therapy Injection. (**A**) 23-gauge standard 3 port vitrectomy; (**B**) Performing core vitrectomy; (**C**) Posterior hyaloid highlighted with intravitreal triescence and lifted with the vitreous cutter; (**D**) Performing peripheral vitreous shave; (**E**) Vitrectomy machine driven delivery of gene therapy with a med-one microdose syringe connected to a DORC 41-gauge subretinal cannula; (**F**) Fluid air exchange; (**G**) Sclerotomy closure.

Current routes of administration include subretinal, intravitreal, and suprachoroidal drug delivery. Subretinal injections bypass the ILM barrier to deliver therapy adjacent to the photoreceptors and retinal pigment epithelium, and allow for localized delivery to the macula, which is the pathological site in AMD, without affecting the rest of the retina [29]. Additionally, there is a lower risk of triggering a systemic immune response due to the immune-privileged location and better shielding of the virus from the immune system. There is also minimal exposure to the vitreous and anterior chamber, which decreases the risk of an immune response [30]. Advances in capsid engineering for subretinal routes, such as geometry-aware capsids, can help expand reach from local injections. For example, the AAV.SPR vector is designed to spread laterally beyond the subretinal bleb, enabling foveal coverage without foveal detachment, and is being evaluated in a trial for X-linked retinoschisis [26]. There are two routes of subretinal delivery: a transscleral route through the choroid, and a transvitreal route (Figure 1), which requires a pars plana vitrectomy and drug injection into the subretinal space [29]. It carries the disadvantage of being a more invasive surgical approach, with associated surgical complications and requiring operating room access.

The suprachoroidal route is a novel route for intraocular delivery, which involves accessing the potential space between the sclera and the choroidal vasculature with targeted and compartmentalized viral vector delivery. Suprachoroidal injection of an AAV8 vector has been shown to enable widespread transgene expression across the RPE and photoreceptors in animal eyes [31]. However, since the suprachoroidal space is adjacent to the highly vascular choroid, there is a potential for rapid egress of the injected material as well as a risk for inducing systemic immune responses [27,29,32]. It carries the advantage of being an in-office procedure.

Intravitreal injections are easy to administer, allowing their use in outpatient clinical settings. The disadvantage of this route is that most AAV subtypes are unable to penetrate the neurosensory retina via this route, due to the barrier effect of the internal limiting membrane (ILM) and vitreous. However, advances in capsid engineering have led to the development of modified AAVs with improved ILM penetration and broader retinal transduction [30,33]. For example, modified AAV vectors withamino acid modification in the heparan-sulfate proteoglycan (HSPG) sites can improve ILM penetration, improving the vectors’ ability to reach the deeper retinal layers [26]. Another disadvantage of the intravitreal delivery route is the potential risk of triggering a systemic immune response with the IVI route, as the drug egresses through the trabecular meshwork and into the systemic circulation [27,29,32], and typically requires pretreatment with corticosteroids.

Gene therapies for nvAMD are aimed at decreasing the levels of VEGF via transduction of anti-VEGF proteins, which does not necessarily require targeting the macula and can be achieved via subretinal, suprachoroidal, or intravitreal delivery approaches. In contrast, GA in dry AMD affects the macula, and current gene therapies aim to target the underlying pathophysiological mechanisms that modulate the complement system, oxidative stress, and metabolic pathways, causing dysfunction of the RPE-Bruch’s membrane complex, choriocapillaris, and photoreceptors in the macula. These features argue that the subretinal and suprachoroidal route may be best suited for dry AMD gene therapies. Current gene therapy trials for both dry and nvAMD are exploring each of the three routes.

## 4. Molecular Mechanisms and Targets for Gene Therapy in Age-Related Macular Degeneration

Gene therapy for AMD involves fundamentally different strategies compared with treatment of inherited retinal diseases. The pathophysiology of AMD involves a complex interplay between genetic susceptibility, environmental factors, and age-associated changes. Unlike gene therapy for inherited retinal diseases, which targets specific monogenic mutations to replace or repair defective genes, gene therapy for AMD aims to modulate disease pathways rather than correct a single genetic defect. The goal of gene therapy in AMD is to transduce cells to enable sustained long-term production of therapeutic proteins such as anti-VEGF agents or complement inhibitors, via the “biofactory approach”, as discussed above.

Several pathophysiological mechanisms play a role in the development of AMD and lead to RPE damage and degeneration, which precede the later stages of AMD, i.e., GA and neovascularization. Oxidative stress is a central driver of disease. The macula is uniquely susceptible to chronic oxidative stress, especially in the metabolically active outer retina, caused by high oxygen consumption, light exposure, and lipid peroxidation [34]. Accumulation of reactive oxygen species leads to the disruption of mitochondrial oxidative phosphorylation, mitochondrial DNA damage, and disruption of autophagy and proteasomal degradation pathways within the RPE [35]. As the phagocytic role of the RPE becomes compromised, cytotoxic lipofuscin deposits accumulate. Accumulation of lipofuscin and toxic bisretinoids further compromises lysosomal function, leading to defective outer segment processing and RPE senescence [35]. Concurrently, age-related alterations in Bruch’s membrane impair nutrient and waste exchange, contributing to drusen formation and outer retinal dysfunction. Inflammation and immune dysregulation play essential roles in AMD. The activation of resident microglia, recruitment of macrophages, and pathological complement system activation create a sustained proinflammatory milieu that amplifies tissue injury [36]. In particular, the complement system appears to play a central role, especially given that genetic variants in the complement pathway have been associated with AMD development, as described below [5]. The complement system is a protein cascade comprised of more than 50 proteins that play a role in the innate immune system. The complement system proteins are activated through a proteolytic cascade by pathogens, debris, or dead cells, either through the classic, alternative, or lectin pathways. Once activated, the complement system promotes inflammation, opsonization (tagging of pathogens or cellular debris with antibodies or complement proteins to enhance their recognition and clearance by phagocytic cells), and direct lysis of cells via the membrane attack complex (MAC) [37]. Some of the earliest evidence of the role of the complement system in AMD pathogenesis was the presence of various complement proteins within drusen [38,39]. In addition to CFH, a number of complement-related genetic variants have been associated with AMD, including C2/CFB, C3, C7, C9, CFI, and SERPING1 [40]. Although the precise mechanisms by which the complement system leads to retinal pathology in AMD are unknown, it is thought that complement dysregulation in AMD exacerbates the chronic low-grade inflammation, oxidative stress, and damage to the RPE cells, choriocapillaris and Bruch’s membrane mentioned above [40,41]. Eventually, inflammatory and metabolic stressors drive upregulation of pro-angiogenic factors such as VEGF, driving pathologic choroidal neovascularization and vascular leakage. Studies have shown that altered telomere length in specific leukocyte subtypes such as T and B lymphocytes may reflect systemic aging processes underlying AMD pathogenesis and could serve as a diagnostic hallmark of this disease. [42]. In light of the above-described mechanisms, current gene therapies for dry AMD involve targeting the complement system. This entails inducing the production of complement inhibitors or modulators. For neovascular AMD, gene therapy aims to produce antiangiogenic factors and VEGF inhibitors.

## 5. Methods for the Selection of Studies

For the purpose of this review, studies in the English language were identified through a comprehensive search of PubMed/MEDLINE, Clinicaltrials.gov, and major ophthalmology conference proceedings, i.e., Association for Research in Vision and Ophthalmology (ARVO). In addition, publicly available information from pharmaceutical and biotechnology company websites was examined to identify ongoing, recently completed, or un-published clinical trials. Eligible studies included completed and ongoing clinical studies, i.e., phase 1, 2, or 3 studies, investigating gene-based therapies for neovascular or geographic atrophy AMD that were registered on Clinicaltrials.gov. Studies that were not registered on this website were not included. Additionally, preclinical studies were not included.

Due to the heterogeneity in study design, phase of study, status of study, outcome measures, and follow-up duration, findings were synthesized qualitatively in a narrative format instead of being analyzed for a systematic review or pooled for a meta-analysis.

## 6. Gene Therapy Clinical Trials for Dry Age-Related Macular Degeneration

Current gene therapy clinical trials for dry AMD aim to combat the progression of GA by targeting the complement system that has been implicated in the pathogenesis of GA. Table 1 lists the current gene therapies being investigated for dry AMD.

In 2017, the first trial for a gene therapy product for GA in dry AMD was conducted by Hemera Biosciences (Waltham, MA, USA). The drug, initially named HMR59, was renamed to JNJ-1887 after acquisition by Janssen Pharmaceuticals (Titusville, NJ, USA). JNJ-1887 (or AAVCAGsCD59) is a recombinant AAV serotype 2 that carries the gene for a soluble form of CD59 under the control of a CAG promotor [43]. It transduces the ganglion cell layer, allowing sustained production of soluble CD59 that diffuses to the outer retina, RPE, and choriocapillaris. The naturally occurring membrane-bound protein CD59 is part of the complement system and acts as a complement regulator. It prevents the complement system from damaging cells by inhibiting formation of MAC. Phase 1 trials (NCT03144999) showed that the drug was well-tolerated [44]. It is currently undergoing a phase 2b randomized, double-masked, multicenter, sham-controlled clinical trial (PARASOL study, NCT 05811351) for GA in AMD [45].

GT005 by Gyroscope/Novartis uses an AAV vector delivered subretinally to induce the expression of complement factor I (CFI) to inhibit complement-mediated cell apoptosis [25,46]. It was evaluated in the FOCUS trial (phase1/2), which was followed by the phase 2 HORIZON (NCT04566445) and the phase 2 EXPLORE (NCT04437368) trials [47,48]. These trials were discontinued by Novartis in 2023 due to lack of treatment efficacy [49].

OCU410 is currently under phase 1/2 trials (NCT06018558) [50]. It involves subretinal injection of an AAV vector carrying the gene for Retinoic-acid related orphan receptor α (*RORA*), which is a nuclear hormone receptor (NHR) that regulates several homeostatic pathways in the retina, such as lipid metabolism, oxidative stress, and inflammation pathways [51]. It is also a known regulator of several AMD genes [52]. VOY-101 is a gene therapy that utilizes an AAV vector injected intravitreally to induce expression of factor H-like protein 1 (FHL-1), which is an alternative splice variant of complement factor H gene, and regulates the complement pathway [53,54]. It is currently in phase 1/2 trials (NCT06087458) [55]. CTx001 is an AAV-based gene therapy that aims to deliver a truncated version of the complement receptor 1 that modulates the complement cascade. The FDA has cleared its Investigational New Drug (IND) application, and the company (Complement Therapeutics, Munich, Germany) is planning a phase 1/2 study (Opti-GAIN) [56,57].

Gene therapy for GA in dry AMD remains in the early exploratory phase. While results from more advanced trials in this domain are not yet available, safety profiles for dry AMD gene therapy have so far been shown to be favorable. Demonstrating efficacy remains challenging, as illustrated by the discontinuation of GT005 despite a strong mechanistic rationale, highlighting the complexity of complement biology in AMD. Additionally, this highlights a broader challenge while developing therapies for GA: demonstrating the slowing down of the progression of atrophy in a very slowly progressive disease requires identifying highly sensitive yet clinically meaningful endpoints and adequate powering. Gene therapy for AMD may need to expand beyond single complement components toward broader regulation of inflammation, oxidative stress, and metabolic pathways, such as those targeted by the OCU410.

## 7. Current Gene Therapy Trials for Neovascular Age-Related Macular Degeneration

Gene therapies for nvAMD are largely focused on inducing anti-VEGF production in retinal cells to ensure durable sustained treatment, with the aim of reducing the burden of frequent anti-VEGF injections. As described above, the pathogenesis of visual loss in nvAMD is related to the production of VEGF by damaged retinal cells, which induces neovascularization. The abnormal new vessels can leak and bleed into the subretinal or intraretinal space. One of the first gene therapy trials for nvAMD was a phase 1 study (NCT00109499) that investigated the intravitreal delivery of a transgene vector of AAV5 carrying pigment epithelium derived factor (*PEDF*) (AdGVPEDF.11D) [58]. PEDF inhibits angiogenesis by binding to and blocking the activity of the VEGF receptor; however, this therapy lacked a clear efficacy signal. Some of the other early gene therapy trials for nvAMD evaluated the transduction of retinal cells to produce sFLT-1 (soluble fms-like tyrosine kinase 1), a potent naturally occurring antiangiogenic protein that binds and inhibits VEGF. A phase 1 study (NCT01024998) conducted between 2010–2014 delivered the gene therapy intravitreally and a phase 1/2 study (NCT01494805) utilized a subretinal route [44,59]. Another early gene therapy trial (NCT013101443) investigated a lentiviral vector that expressed endostatin and angiostatin [60]. Although these therapies were well-tolerated, they did not progress to phase 3 clinical trials due to lack of efficacy and gene expression variability. Other gene therapies for nvAMD that are currently in early phase 1 or 2 trials include NG101, LX102, LX109, KH658, FT-003, EXG102-031, SKG0106, and ABI-110 [61,62,63,64,65,66,67,68]. A few early phase trials are investigating post-transcription silencing of VEGF, VEGF receptors, or hypoxia induced genes [8]. Table 2 summarizes current gene therapies for nvAMD.

The most advanced gene therapies that have progressed to phase 3 clinical trials include ixo-vec, RGX-314, and 4D-150. Ixoberogene soroparvovec (ixo-vec), also known as ADVM-022 or AAV.7m8-aflibercept, is currently in phase 3 trials (NCT06856577, ARTEMIS). It consists of a recombinant AAV vector that induces retinal cells to produce an aflibercept-like protein that binds to and inhibits VEGF. Preliminary results from its phase 1 and 2 trials have been favorable. The OPTIC trial (NCT03748784) was a two-year, open label phase 1 study with a three-year extension (OPTIC-EXT, NCT04645212). It included 30 patients with nvAMD who required frequent anti-VEGF therapy and assigned them to either of two doses, (2 × 10^11^ vector genomes (vg)/eye), or 6 × 10^11^ vg/eye). Results from the two-year OPTIC study showed a reduction in annualized anti-VEGF injections by 80% (10 mean annualized injections to 1.9) and 98% (9.8 mean annualized anti-VEGF to 0.2) in the 2 × 10^11^ vg/eye and 6 × 10^11^ vg/eye cohorts, respectively [69]. Furthermore, almost 50% of patients were injection-free through four years following ixo-vec treatment, and aqueous aflibercept protein levels remained detectable for up to five years after a single injection [70]. Ixo-vec was overall well tolerated. Side effects included inflammation that was dose-dependent and responsive to corticosteroids and did not clinically impact vision [69,70]. The phase 2 LUNA trial (NCT05536973) is an ongoing double-masked, randomized study that includes 60 patients with nAMD randomized across two dose cohorts, 6 × 10^11^ vg/eye or 2 × 10^11^ vg/eye. It is also evaluating multiple prophylactic regimens to reduce intraocular inflammation. Results from this study have shown a decrease in annualized anti-VEGF injection burden of 88% in the 6 × 10^11^ vg/eye cohort and 92% in the 2 × 10^11^ vg/eye cohort at 52 weeks, with 54% (6 × 10^11^ vg/eye) and 69% (2 × 10^11^ vg/eye) of patients remaining injection-free. Best-corrected visual acuity and central subfield thickness remained stable in both cohorts. The safety profile was favorable, and no 6 × 10^11^ vg/eye patients had inflammation at 52 weeks [70]. The phase 3 ARTEMIS trial for this gene therapy is currently enrolling patients, and is planned as a multicenter, double-masked, randomized trial that will evaluate the efficacy of a single ixo-vec injection compared with aflibercept every two to eight weeks in both treatment-naïve and treatment-experienced patients with nvAMD.

Another gene therapy for nAMD that has advanced to phase 3 trials is 4D-150. It consists of a novel AAV viral capsid (R-100) that carries two therapeutic transgenes: a sequence coding aflibercept and a microRNA sequence that inhibits the expression of VEGF-C [71]. The phase 1/2 study of this therapy (PRISM, NCT05197270) showed a favorable safety profile and promising efficacy. At 52 weeks, there was an 83% reduction in annualized injections in the population with severe disease, and an 89% reduction in annualized injections in a broad population [72]. Currently, two phase 3 trials (NCT07064759 and NCT06864988) are actively recruiting subjects [73].

RGX-314 consists of a recombinant AAV8 vector that carries a coding sequence for a monoclonal antibody fragment antigen binding (Fab) protein that mimics ranibizumab. It is currently being investigated in phase 3 clinical trials. It consists of a single subretinal injection delivered by small-gauge vitrectomy. Data from the two-year multicenter phase 1/2a dose escalation study (NCT03066258) included 42 patients that had macular neovascularization secondary to nvAMD, with subretinal or intraretinal fluid in the center subfield [74]. The primary outcome was safety; the study showed that subretinal delivery of RGX-314 was well-tolerated and did not induce clinically recognizable immune responses. Additionally, at two years, participants displayed stable or improved visual acuity and retinal thickness with few or no supplemental anti-VEGF injections required [75]. Additional long-term follow-up data has demonstrated a durable treatment response up to four years [76]. Two phase 3 studies ASCENT (NCT05407636) and ATMOSPHERE (NCT04704921) are actively recruiting subjects [77,78]. A phase 2 study (AAVIATE, NCT04514653) is investigating an alternative suprachoroidal route of delivery for this gene therapy [79].

Another mechanism for gene modulation in nvAMD that has been explored is the delivery of small interfering RNA (siRNA) sequences that target the gene for VEGF or VEGF receptor and prevent the production of these proteins by interfering with the mRNA transcribed from the gene. These therapies included Bevasiranib, ANG211745 (also known as Sirna027) and PF-04523655 (which targeted the RTP801 gene that is activated in the cellular stress response to hypoxia) [80,81]. These were deemed to be safe in early phase trials but were discontinued due to the lack of sufficient efficacy [82]. SYL1801 is an siRNA therapy targeting Notch-Regulated Ankyrin Repeat Protein (NRARP), which has been formulated as an eye drop instead of an intraocular injection. It is being investigated in a phase 2 trial (NCT05637255) in Slovakia, Poland, and Czechia [83].

The only CRISPR/Cas (clustered regularly interspaced short palindromic repeats (CRSIPR)/CRISPR associated proteins (Cas)) based RNA-editing therapy for nvAMD in clinical trials is HG202. It consists of an AAV capsid that delivers a CRISPR/Cas13 RNA-editing therapy that targets and degrades the VEGF-A messenger RNA (mRNA), preventing its expression [84]. It has shown efficacy in mouse models, and provides an option that theoretically avoids permanent changes in host DNA sequence, since it targets mRNA [85]. The first in-human phase 1 trial for this therapy was initiated in China, and recently a phase 1 dose-escalation study (NCT06623279) is underway in the US [86].

In summary, gene therapy for nvAMD has progressed further compared to dry AMD. Although early gene therapy trials (i.e., AdGVPEDF.11D, AAV2sFLT01, rAAV.sFLT-1, and retinostat) for nvAMD established the feasibility and safety of utilizing gene therapy, they failed to show clinically meaningful efficacy, mostly due to variable transgene expression and insufficient anti-VEGF expression. Early trials for post-transcriptional silencing of VEGF through siRNA (e.g., bevasiranib, Sirna027, PF- 04523655) also failed to demonstrate efficacy. However, these studies demonstrated proof-of-concept and paved the way for newer therapies, such as ixo-vec, RGX-314, and 4D-150, that have demonstrated substantial and durable reductions in anti-VEGF injection burden in phase 1/2 studies with acceptable safety profiles, leading to multiple ongoing phase 3 trials.

## 8. Challenges and Limitations of Gene Therapy

Although gene therapy for AMD shows significant promise, several practical challenges exist, including effective gene delivery, efficient transduction, and immunogenicity. Adenoviral vectors are known to provoke a strong immune response, and even though AAV vectors trigger milder effects, the presence of neutralizing antibodies in a patient’s blood may hinder the effectiveness of the gene therapy. Immune responses to the vector may lead to significant ocular and systemic inflammation requiring immunosuppression [28]. The different routes of gene therapy delivery have associated risks. The subretinal delivery approach involves undergoing a pars plana vitrectomy, which is an invasive surgical procedure that can lead to complications such as endophthalmitis, elevated intraocular pressure, retinal detachment, cataracts, epiretinal membrane formation, and macular hole formation [28]. Even though intravitreal and suprachoroidal routes are less invasive, they are still associated with procedure-related risks; the suprachoroidal delivery route also carries a risk of suprachoroidal hemorrhage.

The long-term ocular and systemic safety of gene therapy is not fully understood, and long-term follow-up data is needed to establish safety. Once a gene is introduced into a cell, it cannot be switched off and there is limited control over its expression [87]. Therapies for nvAMD target the VEGF pathway; physiologic levels of VEGF are needed to maintain the normal structure and function of retinal neurons and choroidal vasculature. Unlike conventional anti-VEGF therapy, whose frequency is adjusted based on changing patient needs, gene therapy theoretically produces a sustained suppression of VEGF with unknown long-term effects on the retina and choroid.

Other practical challenges include scaling up drug manufacturing to meet the high demand of nvAMD patients, and setting reasonable drug costs for a lifetime curative therapy while balancing both incentives and patient benefits. These economic considerations may influence the design and feasibility of new therapies [23]. Additionally, before gene therapy can be integrated into clinical practice, guidelines may need to be established to guide appropriate patient selection to provide efficacious and resource-efficient care.

## 9. Limitations of This Study

This review has several limitations. As a narrative synthesis, it does not include a formal pooled or quantitative meta-analysis to establish the efficacy of the gene therapies being discussed. However, given the heterogeneity of gene therapy targets being evaluated in different phases of study, a systematic review or meta-analysis is not feasible at this time. This review only includes studies that are currently registered on ClinicalTrials.gov; hence, studies or trials not registered in this database are not captured. In addition, it does not address or report preclinical investigations exploring additional therapeutic targets. Only English-language sources were reviewed, and non-English conference proceedings, news releases, and company websites were not systematically searched, which may have resulted in the omission of certain studies.

## 10. Conclusions and Future Directions

Gene therapy can revolutionize the way we treat patients with AMD. It has the potential to decrease treatment burden and improve long-term visual and anatomical outcomes. As gene therapy for dry and wet AMD advances into later-stage trials, we remain cautiously optimistic regarding its potential to serve as a safe and durable long-term treatment option. However, retina specialists need to be mindful of the many challenges pertaining to gene therapy, including safety, limitations due to immunogenicity, long-term ocular and systemic side effects, and barriers to drug manufacturing and access. Continued efforts are required to improve precision, safety, and efficacy, including identifying the safest and most effective vectors and delivery routes and minimizing potential adverse effects. In addition, guidelines need to be established for appropriate patient selection before gene therapy can be integrated into clinical practice.

## Figures and Tables

**Table 1:** Gene Therapy Trials for Dry Age-Related Macular Degeneration.

Gene Therapy	Developer/ Sponsor	Mechanism/ Target	Vector	Route of Delivery	Current /Most Recent Phase of Study	Trial ID	Year of Initiation—Year of Completion *	Status	Primary Outcome(s)	Secondary Outcomes	Preliminary Results, If Any
JNJ-1887 (AAVCAGsCD59)	Janssen Pharmaceuticals, Titusville, NJ, USA	Expression of soluble CD59 to inhibit MAC formation	AAV	Intravitreal injection	Phase 2	NCT05811351 (PARASOL)	2023–2026	Active, not recruiting	Change from baseline in square root of GA lesion area in the study eye at month 18 measured by retinal imaging using FAF	Reading speed, BCLLA, retinal sensitivity by mesopic microperimetry, BCVA, NEI-VFQ-25 composite score, FRI index	None
OCU410 (AAV5-hRORA)	Ocugen, Malvern, PA, USA	Expression of RORA that regulates lipid metabolism, oxidative stress and inflammation pathways including CD59	AAV	Subretinal	Phase 1/2	NCT06018558 (ArMaDa)	2023–2026	Active, not recruiting	Safety, change in anatomy of ocular structures, BCVA, LLVA, IOP	Humoral and cellular immune response, shedding of viral vector, serum chemistry	21.4% slower lesion growth from baseline in treated vs untreated fellow eyes, preservation of retinal tissue around GA lesions, stabilization of visual function as measured by LLVA
VOY-101	Perceive Biotherapeutics, San Francisco, CA, USA	Expression of factor H-like protein 1 (complement system regulator)	AAV	Intravitreal	Phase 1/2a	NCT06087458 (JOURNEY)	2023–2028	Recruiting	GA growth rate and change in ellipsoid zone integrity at week 48	Change in BCVA, LLVA and microperimetry; incidence of nvAMD; safety	None
CTx001	Complement Therapeutics, Munich, Germany	Expression of a truncated version of Complement Receptor 1 that modulates the complement cascade	AAV	Not known	Phase 1/2	N/A (Planned: Opti-GAIN)	N/A	IND application cleared, but the trial has not yet started	N/A	N/A	N/A
GT005	Gyroscope (Novartis), Stevenage, Herefordshire, UK	Expression of CFI to inhibit complement-mediated apoptosis	AAV	Subretinal	Phase 2	NCT04566445 (HORIZON)	2020–2024	Terminated	Change from baseline to week 72 in GA	Change from baseline at week 96 in GA, adverse events, BCVA, FRI index, NEI-VFQ-25 composite score, reading performance	No published data. Terminated due to insufficient efficacy

* Estimated or actual. Abbreviations: MAC, membrane attack complex; AAV, adeno-associated virus; GA, geographic atrophy; FAF, fundus autofluorescence; BCLLA, best corrected low luminance visual acuity; BCVA, best-corrected visual acuity; NEI-VFQ-25, National Eye Institute Visual Functioning Questionnaire-25; FRI, functional reading independence; RORA, retinoic acid-related orphan receptor alpha; LLVA, low-luminance visual acuity; IOP, intraocular pressure; CFI, complement factor inhibitor.

**Table 2:** Gene Therapy Trials for Neovascular Age Related Macular Degeneration.

Gene Therapy	Developer/ Sponsor	Mechanism/ Target	Vector	Route of Delivery	Current /Most Recent Phase of Study	Trial ID	Year of Initiation—Year of Completion *	Status	Primary Outcome(s)	Secondary Outcomes	Preliminary Results, If Any
AdGVPEDF.11D	GenVec, Gaithersberg, MD, USA	Expression of PEDF that binds to and inhibits VEGF receptors	Adenovirus	Intravitreal	Phase 1	NCT00109499	2002–2005	Completed	Safety and tolerability	-	Safe and well-tolerated. However, further development was halted due to insufficient efficacy.
AAV2-sFLT01	Genzyme, a Sanofi Company, Cambridge, MA, USA	Expression of sFLT-1 that binds to and inhibits VEGF	AAV	Intravitreal	Phase 1	NCT01024998	2010–2018	Completed	Maximum tolerated dose, adverse events through week 52 and up to 4 years	Decreased retinal thickness through week 52 and up to 4 years	Safe and well-tolerated. However, further development was halted due to insufficient efficacy.
rAAV.sFLT-1	Lions Eye Institute, Perth, Western Australia	Expression of sFLT-1 that binds to and inhibits VEGF	AAV	Subretinal	Phase 1/2	NCT01494805	2011–2017	Completed	Ophthalmic complications, toxicity or systemic complications at 1-month post-injection	Maintenance or improvement of vision without the necessity of ranibizumab re-injections up to 3 years	Safe and well-tolerated. However, further development was halted due to insufficient efficacy.
RetinoStat	Oxford BioMedica, Oxford, UK	Expression of endostatin and angiostatin which are antiangiogenic proteins	Lentiviral vector based on EIAV	Subretinal	Phase 1	NCT01301443 (GEM)	2011–2015	Completed	Incidence of adverse events at 24 weeks	Change in subretinal and intraretinal fluid as measured by OCT at 24 weeks	Safe and well-tolerated. However, further development was halted due to insufficient efficacy.
EXG102-031	Exegenesis Bio, Horsham, PA, USA	Expression of an angiopoietin domain and VEGF receptor fusion protein that binds to and inactivates Angiopoietin-2 and VEGF	AAV	Subretinal	Phase 1	NCT05903794 (Everest)	2023–2026	Active, not recruiting	Frequency, type and intensity of ocular and non-ocular adverse events throughout 52 weeks	Change in BCVA at 52 weeks, average number of supplemental anti-VEGF doses received throughout 52 weeks	None
LX109	Shanghai General Hospital, Shanghai Jiao Tong University School of Medicine, Shanghai, China	Undisclosed	Undisclosed	Intravitreal	Phase 1	NCT06022744	2023–2027	Not yet recruiting	Adverse events at 4 weeks	Change in BCVA and CST, time after LX109 treatment to first salvage treatment, proportion of subjects receiving salvage treatment in study eye, etc. at various time points	None
KH631	Chengdu Origen Biotechnology Co., Ltd., Chengdu, China (In collaboration with Vanotech in the US)	Expression of an anti-VEGF protein (details undisclosed)	AAV	Suprachoroidal	Phase 1	NCT05657301 (VAN-2201)	2023–2027	Recruiting	Incidence of ocular and systemic adverse events, BCVA at 52 weeks	Incidence of ocular and systemic adverse events, rescue injections, BCVA at 104 weeks	None
KH658	Chengdu Origen Biotechnology Co., Ltd., Chengdu, China (In collaboration with Vanotech in the US)	Expression of an anti-VEGF protein (details undisclosed)	AAV	Suprachoroidal	Phase 1	NCT06825858 (VAN-2401)	-	Not yet recruiting	Incidence of ocular and systemic adverse events, rescue injections at 24 and 52 weeks, BCVA at 52 weeks	Change in BCVA at 24 weeks	None
BD311	Shanghai BDgene, Shanghai, China	Expression of an antibody that binds to and inactivates VEGFA	Lentiviral vector (IDLV)	Suprachoroidal	Phase 1	NCT05099094	2021–2025	Active, not recruiting (in China, no US sites yet)	Adverse effects at multiple time points up to 12 months	Changes in macular intraretinal fluid, subretinal fluid, CRT, area of CNV, area of fluorescein leakage, number of rescue treatments, BCVA at multiple time points up to 12 months	None
HG202	HuidaGene Therapeutics Co., Ltd., Shanghai, China	CRISPR/Cas13 based system that targets and degrades VEGFA mRNA, preventing its expression	AAV	Subretinal	Phase 1	NCT06623279 (BRIGHT)	2025–2031	Not yet recruiting	Incidence and severity of ocular and systemic adverse events at 52 weeks	Mean change in BCVA at 52 weeks, mean change in annualized rate of supplemental injections at 52 weeks	None
SKG0106	Skyline Therapeutics, Shanghai, China	Expression of Vb24, an anti-VEGF protein	AAV	Intravitreal	Phase 1/2a	NCT05986864	2024–2026	Recruiting	Dose-limiting toxicities at 4 weeks; type, severity and incidence of ocular and systemic adverse events at 52 weeks	Change in BCVA, CST and NEI-VFQ-25 score at each visit through 52 weeks	None
ABI-110	Avirmax Biopharma Inc., Hayward, CA, USA	Expression of VEGF-Trap, a recombinant protein analogous to aflibercept	AAV	Intravitreal	Phase 1/2	NCT0655001	2024–2026	Recruiting	Incidence of treatment-emergent adverse effects at 52 weeks	Change in BCVA at 52 weeks, immunogenicity, pharmacodynamics and pharmacokinetics, optimal dose	None
NG101	Neuracle genetics, Inc., Seoul, Republic of Korea	Expression of aflibercept that binds to and inhibits VEGF	AAV	Subretinal	Phase 1/2a	NCT05984927	2023–2030	Recruiting	Incidence of ocular and systemic adverse events at 24 weeks	Systemic immunogenic response, CST, BCVA, cumulative number of rescue therapy injections at various time points	None
RRG001	Shanghai Refreshgene Technology Co., Ltd., Shanghai, China	Undisclosed	AAV	Subretinal	Phase 1/2a	NCT06141460	2023–2030	Recruiting (in China, does not have US IND yet)	Incidence of ocular and systemic adverse events at 52 weeks	Change in BCVA and CRT at 52 weeks	None
FT-003	Frontera Therapeutics, Bedford, MA, USA	Expression of a recombinant fusion protein analogous to aflibercept	AAV	Intravitreal	Phase 1/2	NCT06492863	2023–2028	Recruiting (in China, received IND clearance in the US in 2024)	Incidence of adverse events at 12 weeks	BCVA and OCT change at week 24	None
LX102	Innostellar Biotherapeutics Co., Ltd., Shanghai, China	Expression of VEGF-Trap, a recombinant protein analogous to aflibercept	AAV	Subretinal	Phase 2	NCT06196840 (VENUS)	2024–2029	Active, not recruiting (in China, does not have US IND yet)	Mean change in BCVA at 36 weeks	Mean change in BCVA 52 weeks, mean change in CST at 36 and 52 weeks, durability of LX102 at 52 weeks, incidence of ocular and systemic adverse events at 36 and 52 weeks	None from phase 2, but data from a prior phase 1 study evaluating the intravitreal route of delivery showed a favorable safety profile
SYL1801	Sylentis, S.A., Tres Cantos, Spain	Inhibition of the expression of the *NRARP* gene which is involved in angiogenesis	siRNA formulation	Topical drops	Phase 2	NCT05637255	2022–2024	Unknown status	Change in BCVA on day 42	Proportion of subjects who maintained visual acuity, proportion of subjects who needed rescue medication, change from screening on leakage area, adverse events, etc. through day 42	Low incidence of treatment-emergent adverse events.
AGN211745 (Sirna-027)	Allergan, Dublin, Ireland	Inhibition of the expression of the *VEGF-Receptor 1* gene	Naked siRNA	Intravitreal	Phase 2	NCT00395057	2007–2009	Terminated	Percentage of patients with improvement in BCVA of 15 or more letters at month 3	Lesion size assessed by FA and photograph, foveal thickness, and NEI- VFQ at month 3; time to treatment with standard of care at month 6	Terminated due to lack of sufficient efficacy
PF-04523655	Quark Pharmaceuticals, Fremont, CA, USA	Inhibition of the expression of the hypoxia-inducible gene *RTP801*	Naked siRNA	Intravitreal	Phase 2	NCT00713518 (MONET study)	2009–2011	Completed	Mean change in BCVA by week 16	Incidence and severity of ocular and systemic adverse events, change in CST, etc.	Further development of the drug was halted due to only modest efficacy
Bevasiranib	OPKO Health, Inc., Miami, FL, USA	Inhibition of the expression of the *VEGF* gene	Naked siRNA	Intravitreal	Phase 3	NCT00499590 (COBALT)	2007–2009	Terminated	Avoidance of 3 or more lines of vision loss at week 60	Need for rescue therapy, time to rescue therapy through week 60	Despite favorable results from early-phase trials, this was terminated due to lack of sufficient efficacy
Ixo-vec (ADVM-022)	Adverum Biotechnologies, Inc., Redwood City, CA, USA	Expression of an aflibercept-like protein that binds to and inhibits VEGF	AAV	Intravitreal	Phase 3	NCT06856577 (ARTEMIS)	2025–2030	Recruiting	Mean change in BCVA at weeks 52 and 56	Mean number of aflibercept injections received, percentages of participants with worsened and improved BCVA, mean change in CST, time to dry retina, change in NEI-VFQ-25, etc. all through week 56.	None from phase 3. Prior phase 1/2 data showed favorable safety and about 86% reduction in injection burden up to 4 years.
4D-150	4D Molecular Therapeutics, Emeryville, CA, USA	Expression of an aflibercept-like protein that binds to and inhibits VEGF, as well as a microRNA sequence that inhibits the expression of VEGF-C	R-100 (novel AAV capsid)	Intravitreal	Phase 3	NCT07064759 (4FRONT-2), NCT06864988	2025–2029, 2025–2028	Recruiting	Mean change in BCVA at week 52	Mean annualized number of aflibercept injections received, proportion of subjects not requiring aflibercept injections after week 4, mean change in CST, etc., all through weeks 52 and 104.	None from phase 3. Prior phase 1/2 data showed favorable safety and about 83% reduction in injection burden at 1 year
RGX-314	AbbVie, North Chicago, IL, USA	Expression of a ranibizumab-like Fab fragment that binds to and inactivates VEGF	AAV8	Subretinal	Phase 3	NCT05407636 (ASCENT), NCT04704921 (ATMOSPHERE)	2022–2027, 2020–2027	Recruiting	Mean change in BCVA at week 54, incidence of ocular and systemic adverse events at week 50	Proportion of participants with <2 supplemental anti-VEGF injections through week 54, proportions of participants with worsened or improved BCVA through weeks 54 and 98, mean change in central retinal thickness through weeks 54 and 98, percent reduction in anti-VEGF annualized rate through weeks 54 and 98, etc.	None from phase 3. Prior phase 1/2 data showed favorable safety and a reduction in injection burden at 2 years

* Estimated or actual. Abbreviations: PEDF, pigment epithelium derived factor; VEGF, vascular endothelial growth factor; AAV, adeno-associated virus; sFLT-1, soluble fms-like tyrosine kinase 1; EIAV, equine infectious anemia virus; BCVA, best-corrected visual acuity; CST, central subfield thickness; NEI-VFQ-25, National Eye Institute Visual Functioning Questionnaire-25; IND, investigational drug application; IDLV, integration-deficient lentiviral vector; CRT, central retinal thickness; CNV, choroidal neovascular membrane; CRISPR/Cas, clustered regularly interspaced short palindromic repeats/CRISPR-associated proteins; OCT, optical coherence tomography; NRARP, Notch-Regulated Ankyrin Repeat Protein; FA, fluorescein angiogram.

## Data Availability

No new data were created or analyzed in this study. Data sharing is not applicable to this article.
